# Impact of collaborative learning on student engagement in college English programs: mediating effect of peer support and moderating role of group size

**DOI:** 10.3389/fpsyg.2025.1525192

**Published:** 2025-04-09

**Authors:** Hong Li

**Affiliations:** Public Foreign Language Department, Xinzhou Normal University, Shanxi, China

**Keywords:** collaborative learning, engagement, peer support, group size, English program, public colleges

## Abstract

Collaborative learning (CL) and student engagement have been extensively researched. There are few studies on the interaction between college students and its underlying mechanism. This research investigates how CL activities assist in boosting student engagement in English programs at public sector colleges. A purposive sampling of 425 intermediate students from a Chinese public college was used to evaluate the direct impact of CL on engagement, the mediating effects of peer support and the moderating influence of group class using structural equation modeling (SEM). The findings show that CL positively relates to peer support but not students’ engagement, and the relationship between peer support and engagement is also significantly positive. However, the specific indirect impact indicates that specific peer support mediates CL activities and students’ engagement to a whole extent. Our results underscore CL’s potential to improve students’ engagement. The results indicated that peer support increases students’ engagement when the group size is less than others. This study is significant for instructors, administrators, educators, policymakers, students, and researchers seeking effective techniques to enhance student engagement in English programs and learning.

## 1 Introduction

Certainly, English language instructors strive for student engagement in their courses (Peng, 2021). This focus is not distant from Chinese education, where the college curriculum encourages English program professors to use active pedagogy to promote students’ engagement in language learning and meaningful learning in a social environment. Peer support may be socially distinctive in China since it emphasizes interpersonal collaboration, communication, and collective harmony (Guo et al., 2024). Therefore, Chinese youngsters esteem their great peers and expect to copy them. As a result, Chinese students must prioritize peer support. Engaging students in a Chinese environment might be tough due to many educational challenges. In China, teacher-centered teaching remains a significant issue. McMahon et al. (2023) research on recognizing university students’ metaphors suggests that pedagogical students usually took a passive part in their education. This viewpoint is diametrically opposite to China’s educational focus on communication skills. According to Zhang and Liu (2023), the English program’s emphasis shifted to standardize testing due to unmet national expectations for intermediate user proficiency. Student-centeredness may be absent from the Chinese classroom because educators must address several issues, leaving little time and space for innovation (Bui, 2022). According to research studies (Liu J. et al., 2022; Sun, 2022), Chinese instructors encounter major obstacles such as disobedience and aggression, which jeopardize optimum class delivery and, as a result, the execution of tactics focused on students. Classroom size is another educational problem that may impede student-centered instruction.

According to Guo et al. (2019), the average number of students per classroom in China is 25, with a high of 50 in metropolitan institutions. According to Li and Li (2021), reducing big classrooms is critical to improving China’s education quality, classroom didactics, and classroom management. Although the Chinese government claims a continuing class-size policy of no more than 35 students per classroom, no modifications have happened (Shan, 2020). The research setting for this study was not an exception to the educational concerns outlined above, more notably in terms of classroom size and teacher-centeredness. As a result, for this study, it was critical to (i) take a reflective role in our teaching context to bring changes to the classroom, (ii) promote learners’ engagement to meet the pedagogical orientations of the national curriculum, and (iii) choose an approach or method that is likely to be implemented in our context. In this attempt, collaborative learning (CL), a student-centered strategy to achieve a shared objective, developed from a literature analysis that indicated diverse advantages for educational communities in different circumstances (Shehata et al., 2024). This strategy also fulfilled our goal of increasing students’ engagement. CL enhances college education (CE) by boosting cognitive, motivational, and social outcomes. This research defines CL as a combination of teaching and learning practices that enable students to collaborate, achieve shared objectives, seek mutually beneficial results, discuss materials, assist each other comprehend ideas, and promote hard effort.

However, the evidence suggests that students confront challenges that impact the effectiveness of CL (e.g., uneven individual engagement in group assignments, lack of communication skills, and incapacity to collaborate with peers) (Lai, 2021). Teachers have challenges in planning and managing CL activities, including inadequate group assignments, poor time management, and ineffective monitoring of collaborative activities (Zydziunaite et al., 2020), which continue to be a topic of public discussion. Given that no comparable studies concentrated on CL and students’ engagement in a Chinese environment at the time of the inquiry, our study was exploratory. The study goal was to introduce CL and see how it affected the engagement of our college students in a southwest area of China. To achieve this purpose, we have submitted the following study questions: Is there a connection between CL and students’ engagement? Is there a link between CL and peer support? Is there a connection between peer support and students’ engagement? What influence will CL assignments have on students’ engagement in the English program? Is class size important?

This study want to fill this gap by investigating how CL may affect students’ engagement. This might provide further insight into whether CL enhances students’ engagement via peer support systems. Furthermore, although past research has examined CL and peer support, the impact of group size on student engagement remains little explored. Although CL and peer support have been extensively researched concerning student engagement, there is a paucity of studies investigating the moderating effect of group size on this relationship. Previous research suggests that group size might affect participation, interaction quality, and learning dynamics. Nonetheless, empirical information regarding the impact of group size on the efficacy of peer support in augmenting student involvement is insufficient. This research examines the moderating effect of group size on the connection between peer support and student engagement within a CL context.

## 2 Related work and hypotheses development

### 2.1 Collaborative learning and students’ engagement

In a word, collaborative learning is defined as learning via interaction (Strauß and Rummel, 2020). According to Herrera-Pavo (2021), CL involves group collaboration to attain shared objectives. According to Wu (2023), learning occurs via learner interactions and negotiations. Completing a specific assignment encourages this socializing by allowing students to support one another while developing their academic talents and group work capabilities (Romanow et al., 2020). CL has an emotional impact and enhances learners’ academic lives by promoting communication, listening, and respect for diverse views and opinions (Dyson et al., 2021). While CL and cooperative learning are sometimes interchangeable, Han and Ellis (2021) point out a theoretical distinction. Cooperative learning involves students working in groups organized by the teacher, who retains control and decision-making as students advance with their work and peers (Olaya and González-González, 2020). CL, on the other hand, indicates that students work independently, discovering and growing their own knowledge while the instructor watches and offers feedback on their projects (Yang, 2023). Cooperative learning fosters group work under the teacher’s leadership, while CL encourages students to organize and make decisions for themselves (Gigauri and Khan, 2025). Lauermann and Berger (2021) suggests that students take responsibility for both their own and others’ learning, leading to increased engagement. CL improves engagement, a vital component. Computer-mediated collaborative work increases student engagement and motivation to interact with course material.

Collaborative learning may increase student engagement by employing ideas, sharing, and comprehending other points of view. Proper cognitive processes improve students’ learning and academic achievement (Reeve et al., 2020). Qureshi et al. (2023) found that CL enhances curricular commitment, offers resources, and facilitates knowledge transfer. A CL environment promotes engagement and produces a positive learning environment. Luan et al. (2023) found that students’ beliefs influence peer support, influencing engagement. Wen (2021) states that students play an important part in establishing CL in the classroom as an engagement strategy. There is little benefit in CL exercises if just one student performs all of the effort. CL activities are effective when all group members complete and accept responsibility for their roles (Herrera-Pavo, 2021). Proponents of CL argue that working together develops social skills and individual responsibility when learners commit to a common objective (Fernandez-Perez and Martin-Rojas, 2022). It should be highlighted that instructors also play an important role as they design, prepare, and build a collaborative atmosphere that encourages motivation and engagement. In other words, CL has a “deliberate” significance since activities performed by students are specially designed by teachers for pairs or small groups (Warsah et al., 2021), hence positively impacting students’ learning experiences. To improve group work, instructors must emphasize the importance of each student by allocating roles to all group members. In this regard, Rusticus et al. (2023) found that students had unfavorable opinions of CL because certain group members assumed the majority of the labor. These results emphasize the importance of group arrangement and role distribution in ensuring active participation and commitment from all students. Empirical investigations have shown a variety of other CL advantages. There is evidence that in collaborative-based work, students’ focus was shifted away from their grades and toward self-satisfaction as a consequence of group effort on difficult assignments. Furthermore, given the social character of learning, many CL research focus on the development and consolidation of learners’ social skills.

Xu et al. (2023) research found that cooperatively analyzing, synthesizing, and assessing problems led to considerable improvements in higher-level thinking abilities. Research on the influence of CL on instructors has shown positive benefits in widening their teaching competencies. According to Tzenios (2020), educating teachers about the benefits of different teaching styles for students’ learning is crucial. Thus, the hypothesis is:

*H1: Collaborative learning positively and significantly influences the students’ engagement in the English program.*

### 2.2 Collaborative learning and peer support

Collaborative learning is an educational method that fosters activities that encourage students to work together to accomplish a shared objective (Strauß and Rummel, 2020). As a consequence, their relationships and reliance grow. According to one perspective (Han et al., 2020), learning is a social activity where peer contact is important for cognitive growth and knowledge building. When students collaborate, they participate in shared discussions, solve issues, and complete cooperative activities, resulting in greater mutual understanding and better social relationships. Empirical research has demonstrated that CL settings dramatically improve student peer support (Er et al., 2021). For example, language learning research has shown that collaborative assignments such as group discussions, peer review sessions, and joint presentations help students establish trust and enhance their communication abilities. Finally, these activities lead to greater support among students. Through CL, students depend on one another for feedback, emotional support, and academic aid, which develops their peer support networks (McLaughlin and Sillence, 2023).

Furthermore, collaborative tactics are especially beneficial in English language programs because they allow students to practice language skills interactively and engagingly (Su and Zou, 2022). This promotes strong peer relationships and generates a supportive school climate that improves learning. Cultural variables also contribute to the validity of this hypothesis. The collectivist culture of Chinese public universities, which emphasizes social harmony and group cohesiveness, provides further support for peer interactions (Liu M. et al., 2022). The focus on group objectives is consistent with CL approaches, which foster collective accomplishment and mutual support. Thus, based on this logic, it is possible to deduce that when CL activities increase, students’ peer support for one another is likely to expand, and the following hypothesis is proposed:

*H2: Collaborative learning positively and significantly influences students’ engagement in English programs.*

### 2.3 Peer support and students’ engagement

It has been proven that there is a link between social environment and academic engagement. The self-determination theory suggests that people’s psychological needs are met via organic interactions with their social environment (Zhang et al., 2024). Given that social engagement was a component of the academic environment, it made theoretical sense that the degree to which social support satisfied students’ psychological needs would affect academic engagement (Karimi and Sotoodeh, 2020; Ma W. et al., 2024).

The positive impact of peer support on academic engagement has been supported by cross-sectional and longitudinal empirical investigations (Bradley et al., 2021). Peer support and encouragement from college students would boost academic engagement and achievement (Tao et al., 2022). However, little empirical research has explicitly examined the connection between peer support and academic engagement among college students (Amerstorfer and Freiin von Münster-Kistner, 2021). Peer support is receiving social and emotional support from peers based on mutual respect, shared responsibility, and agreed-upon helpfulness. Peer support is a concept that has been extensively researched in mental health services and is taken into account in workforce policy-making (Fan et al., 2024). In education, peer support refers to the help and support that students provide one another while they are learning.

Peer support is essential for students’ emotional and academic growth. It significantly reduces learning anxiety by allowing students to share their triumphs, anxieties, interests, and concerns (Peltier et al., 2022). Peer support improves feedback and communication, leading to excellent CL possibilities in the academic setting (Mora et al., 2020). Peer support increases motivation, develops self-regulated learning abilities, and mediates the link between teacher support and students’ self-efficacy. The positive effects of peer support on language learning engagement, language learning fatigue, and positive language emotions have been documented in the context of English language programs, despite the small number of research (Wang et al., 2024). Using peers as learning agents leverages their inherent ability to increase engagement.

To make peer support tactics more successful, it is crucial to understand the processes by which peer support mechanisms affect student engagement (Bradley et al., 2021). According to developmental and educational psychologists, peers may impact cognitive development in two ways: (a) as natural instructors and (b) as contributors to task orientation, perseverance, and incentive to succeed (Lin et al., 2024). From preschool until grade school, children may learn and teach from their peers via various social interactions. Vygotsky and Piaget, among other developmental theorists, emphasized the significance of social connections in cognitive development (Rubtsov, 2020). Vygotsky proposed that peer-size social interactions are rich in useful information and skill exchanges (Ghavifekr, 2020) that help children adapt their cognitions by interacting with peers who share their developmental level but may have a different language, behavioral styles, and opinions. Piagetian theory suggests that self-examination leads to improved thinking and learning and internalization of academic objectives (Li, 2023). Peer tutors may get better results than their students. Providing explanations helps explainers retain knowledge more effectively (Lachner et al., 2021). Providing and getting detailed assistance and answers is linked to improved math skills. Research suggests that reciprocal teaching and learning might lead to cognitive advances for students (Ma H. et al., 2024).

Additionally, peer interactions promote engagement. According to Wentzel et al. (2021) research, students’ social ties with peers significantly impact their motivation and academic achievement. Consistent signals about academic achievement from social influencers might encourage students to adopt these values and pursue positive academic objectives. The ideals and expectations imposed on students in peer and school environments may lead to school disengagement.

Based on the given literature, we propose the following hypothesis:

*H3: Peer support positively and significantly influences students’ engagement.*

*H5: Peer support mediates the relationship between collaborative learning and students’ engagement.*

### 2.4 Moderation effect of group size

Previous research on the impact of group size on performance has been conducted in laboratory and corporate settings using a social-cognitive paradigm in social psychology (Youssef et al., 2023). Research has shown conflicting results in performance, participation distribution, conformance, and satisfaction. There is controversy concerning the lowest number of persons in a group, with some scholars defining two individuals working together in a dyad as the least group size (Leib et al., 2021), while others claim that a group is formed of three or more people. The definition of group size is ambiguous, with some research requiring four or more participants and others limiting it to two or three (Hennig-Thurau et al., 2023). According to social psychology research, individuals working in dyads outperform those in triads, bigger groups of four or more, or individuals working alone. Increasing the number of members in a group may diminish motivation and effort to work jointly on a task, exhibiting a social loafing effect in big groups. The research found that group performance dropped with increasing group size and task complexity whether participants solved tough intellectual issues alone or in same-sex groups of 2, 3, 6, or 10 (Corrégé and Michinov, 2021; Sarsons et al., 2021). They discovered that work groups of three to eight persons are more productive than groups of nine or more. Yetton and Bottger’s (1983) investigation of 87 groups of two to six individuals doing a collaborative activity indicated that performance did not increase for groups bigger than four. Research on social dilemma problems has shown that cooperation decreases as group size increases. A study of three- and seven-member groups (Chao et al., 2021) discovered that smaller groups were more cooperative than bigger ones.

Research suggests that bigger group sizes may boost performance in certain activities. Corrégé and Michinov (2021) found that four-member groups outperformed dyads on a memorizing assignment under cooperative conditions but not competitive ones. Li and Tang (2024) discovered that four-member groups outperformed dyads in identifying an item via a sequence of questions, with fewer failures and less time spent on each issue. However, dyads outperformed people working alone on the same criterion. Erlangga (2022) research divided students into groups of 1, 2, 5, or 10 o answer multiple-choice problems. They discovered that group performance improved as group size increased. Several studies found similar results in solving highly intellectual issues (letters-to-numbers) in groups of two, three, four, or five persons—groupings of more than three outperformed dyads and the top individual in “nominal” groupings. To reconcile inconsistent data on the influence of group size on performance, it is important to evaluate the job at hand. When a group member provides an accurate answer with great demonstrability, the group surpasses the best individual. Research suggests that larger group sizes lead to worse performance on low-demonstrability issues, as members fail to identify the right solutions provided during discussions. Thus, the hypothesis is:

*H4: The group size moderates the relationship between peer support and student engagement.*

**FIGURE 1:**
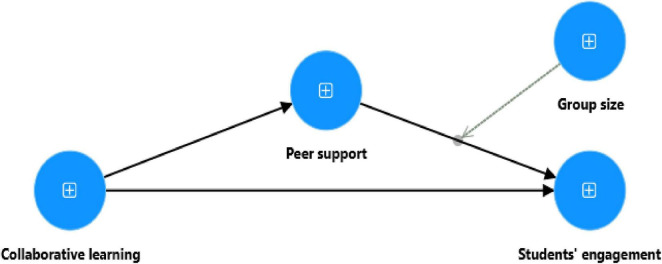
Conceptual collaborative learning model.

Thus, the proposed model (Figure 1) is as follows:

## 3 Methodology

### 3.1 Research design

#### 3.1.1 Research design and rationale

The research used a quantitative, cross-sectional survey design with a time-lagged data collection method. A total of 425 students enrolled in English programs at Chinese public institutions provided data at three different time points. This design was selected for a variety of reasons. First, a quantitative approach enabled us to use standardized, pre-validated questionnaires to assess the dimensions of CL, peer support, group size, and student engagement. This permitted empirical association testing using structural equation modeling (SEM) and SMART PLS 4. Second, the time-lagged design was used to reduce any common method bias by temporally separating the measurements of independent, mediating, and dependent variables. Finally, the cross-sectional survey allowed for fast data collection from a large sample, which increased the robustness and generalizability of our results within the intended educational setting. Overall, this approach offered a good methodological foundation for investigating the intricate interactions between the research variables.

### 3.2 Data collection and sampling procedure

This research included students in their second year of intermediate programs and was ethically approved by the specified educational institutions. All participants provided informed permission and were guaranteed their identity and confidentiality. We created a questionnaire and gave codes based on students’ last four digits of their school registration numbers. A 3-week time lag between data collection sessions allowed us to match students’ replies and eliminate method bias in self-report measures (Podsakoff et al., 2003). At time 1 (week 5, with a time lag of 3 weeks), students completed and submitted the questionnaire to their professors after lessons, resulting in 230 copies. Students returned to their professors at time 2 (week 9), completing the same questionnaire as at time 1. At time 3 (week 14), students completed the identical questionnaire used in time 1 and time 2 and submitted it to their professors after lessons. We deleted 41 copies of the questionnaire with inconsistent replies from time 1, time 2, and time 3. The resulting dataset included 425 (89% matched) answers from the time-lagged sample. The study included students from five China public colleges. The study included 255 (60%) men and 170 (40%) girls aged 21–27. The demographic detail (Table 1) is as follows:

**TABLE 1:** Demographics profile.

College	Total (*n*)	Men (*n*, %)	Women (*n*, %)	Age 21–23 (*n*, %)	Age 24–27 (*n*, %)
A	100	60 (60%)	40 (40%)	70 (70%)	30 (30%)
B	100	60 (60%)	40 (40%)	75 (75%)	25 (25%)
C	80	45 (56.3%)	35 (43.8%)	20 (25%)	60 (75%)
D	75	40 (53.3%)	35 (46.7%)	15 (20%)	60 (80%)
E	70	50 (71.4%)	20 (28.6%)	45 (64.3%)	25 (35.7%)
Total	425	255 (60%)	170 (40%)	225 (52.9%)	200 (47.1%)

### 3.3 Measurements

To confirm the reliability of the measurements, this study use well established scales that have been previously verified in the literature. Before the main study, this study conducted a pilot test of these instruments with a small cohort of 30 students to evaluate clarity, cultural suitability, and contextual relevance. The feedback from this pilot study, together with professional evaluation from professors in educational measurement, validated the content of the scales. The primary study assessed internal consistency using Cronbach’s alpha coefficients, resulting in values of 0.87 for CL, 0.89 for peer support, and 0.91 for student engagement, demonstrating strong reliability.

#### 3.3.1 Collaborative learning

This study adapted an 8-item CL scale based on an extant literature review following the scale development procedures (Okolie et al., 2022). Responses ranged from 1 equal to disagree and 7 to strongly agree strongly. A sample item includes: *I feel that the group task helps me reflect better ways to work together.*

#### 3.3.2 Peer support

The Peer Support Scale is based on the Peer Assets Scale, a two-dimensional scale with six elements (Hanson and Kim, 2007). Many research studies have presented it as a single dimension with four components (Yang and Xiang, 2024). The sample question “My friends often discussed problems related to study with me” was assessed on a 7-point Likert scale (1 = strongly disagree, 7 = strongly agree), with higher average scores suggesting greater levels of peer support. In previous studies, this scale was employed with college students.

#### 3.3.3 Students’ engagement

The Student Engagement in College Items, which has 28 items overall and three dimensions, served as the basis for the Engagement Scale (Freng, 2020). Followed studies given that the scale does not identify specific dimensions and that too many things might dull students, this study kept some sample items from each dimension, and the new scale utilized in this research consists of four items (for example, “I look forward to learning every day”) that was used by Yang and Xiang (2024). The items were assessed on a 7-point Likert scale (1 = strongly disagree, 7 = strongly agree), with higher average scores indicating better levels of student engagement. This scale has been frequently utilized among college students in previous studies.

## 4 Analytical strategy

The research uses three-step SEM to verify the findings and evaluate the theories put forward. Confirmatory factor analysis (CFA) verified the model’s quality in the first phase. The product’s quality is validated by evaluating its reliability, item loadings, discriminant and convergent validity. Table 4 shows the item loading, composite reliability, and average variance extracted (AVE). To validate discriminant validity, the inter-correlation between variables must be less than the square root of AVE.

Furthermore, the influence of CL on students’ engagement must be investigated via peer support and group size moderation. We then investigate the structural impacts. The major goal of this study is to investigate the direct and moderating impacts of CL on engagement and peer support. The specific indirect impact is calculated in the third and final phases to create the findings, and Smart-PLS was used.

### 4.1 Descriptive statistics

To assess the distribution and reliability of the measurement instruments, descriptive statistics, including minimum, maximum, mean, standard deviation, skewness, and kurtosis, were computed for CL, peer support, and student engagement, which are summarized in Table 2.

**TABLE 2:** Descriptive statistics.

	Min	Max	Mean	Standard deviation	Skewness		Kurtosis	
	Statistic	Statistic	Statistic	Statistic	Statistic	Standard error	Statistic	Standard error
CL	1.000	7.000	5.918	1.036	1.701	0.118	3.489	0.236
PS	1.000	7.000	5.453	1.156	0.872	0.118	0.734	0.236
SE	2.000	7.000	6.294	0.895	2.181	0.118	5.660	0.236

CL, collaborative learning; PS, peer support; SE, student engagement.

These findings show that all variables fall within an acceptable range of normality. The skewness and kurtosis results show that the distributions are somewhat non-normal but still fall below acceptable limits for SEM. These figures support the validity of the study’s measuring tools.

### 4.2 Data analysis and results

Before doing the structural analysis, the present inquiry used a two-phase data analysis technique. An investigation of the outer loadings and reliability followed the CFA validation of the PLS measurement model. The final step evaluates the convergent and discriminant validity of all variables. The outer loadings indicate the sequence in which each item is loaded into its associated factor, with a minimum threshold of 0.7. As a result, each of the constructions fulfilled the minimal criteria of 0.7. The three constructions’ item loading, reliability, and AVE were all investigated except for group size. As a result, all of the builds meet the basic criteria. The AVE value, which must be more than 0.5, confirms the validity of the convergent hypothesis. The 0.5 requirements suggest that the constructs’ variance may be traced to specific items. As a result, all of the constructs tested in this study exceeded the minimal criterion of 0.5, as shown in Table 3. According to Cheung et al. (2024), composite reliability is the most effective measure for determining the internal consistency of conceptions. A composite reliability level of 0.7 is the absolute minimum.

**TABLE 3:** Outer loadings, reliability, and validity.

	Outer loadings	Composite reliability	AVE
**Collaborative learning**			
CL1	0.915		
CL2	0.931		
CL3	0.857		
CL4	0.855		
CL5	0.917		
CL6	0.932	0.963	0.813
**Peer support**			
PS1	0.822		
PS2	0.861		
PS3	0.774	0.860	0.672
**Students’ engagement**			
SE1	0.844		
SE2	0.865		
SE3	0.828		
SE4	0.881	0.916	0.731

Furthermore, discriminant validity must be verified throughout data analysis. It demonstrates the distinctness of each concept. To confirm discriminant validity, the square determinant of the AVE for a specific construct must be greater than the correlation of all other constructs. Table 4 shows that all variables have discriminant validity since the diagonal value (square determinant of AVE) is bigger than the bivariate correlation of other constructs. As a consequence, the model demonstrates discriminant validity.

**TABLE 4:** Discriminant validity.

	Collaborative learning	Group size	Peer support	Student engagement
Collaborative learning	0.902			
Group size	0.315	1.000		
Peer support	0.442	0.143	0.820	
Student engagement	0.364	0.284	0.465	0.855

The data for this study is cross-sectional, can sometimes face the issue of multicollinearity. To address this biasness, the study collected data across three different time frames to avoid the biasness of respondents. Additionally, this study checked for multicollinearity using the variance inflation factor (VIF). The VIF values are below the threshold as shown in Table 5, which confirms that there are no issues of bias or multicollinearity in our data.

**TABLE 5:** Multicollinearity.

	VIF
Collaborative learning	1.364
Group size	1.130
Peer support	1.258

This study evaluated the adequacy of the proposed model using important model fit indices such as SRMR, d_ULS, d_G, Chi-square, and NFI in the Table 6. The SRMR values (0.07) suggest an adequate model fit, since values less than 0.08 are often deemed appropriate. The NFI values (0.751) show a fair model fit, which supports the structural validity of our suggested model. Chi-square values also demonstrate the model’s resilience, with the estimated model fitting better than the saturated model. The last procedure required verifying the proposed model using the goodness of fit formula. First, the R-square are poor for peer support and moderate for students’ engagement. The *Q*^2^ for both predictive are above the zero threshold values. Therefore, the overall model is fit (Table 7).

**TABLE 6:** Model fit.

	Saturated model	Estimated model
SRMR	0.072	0.07
d_ULS	0.544	0.519
d_G	2.983	2.974
Chi-square	3,274.482	3,248.918
NFI	0.747	0.751

**TABLE 7:** R-square.

	R-square	R-square adjusted	*Q* ^2^
Peer support	0.195	0.193	0.126
Student engagement	0.332	0.325	0.229

### 4.3 Structural paths

**FIGURE 2:**
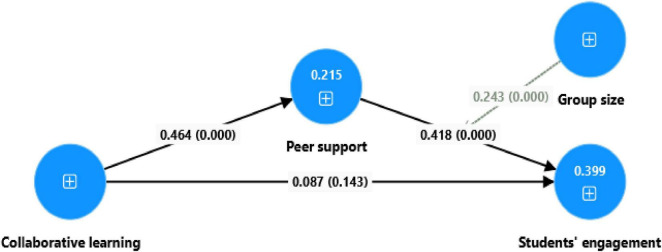
Direct paths.

To test if the path coefficient contributed to the model’s statistical significance, the present research used 5,000 bootstrapping with 425 participants. Tables 8, 9 and Figure 2 contains all of the structural model estimates. Table 8 presents the study’s direct hypothesis, which reveals that all hypotheses are strongly supported at the 99% confidence level, except the CL to students’ engagement, which is considered significant at the 90% level. The first direct positive correlation between peer learning and collaborative support (β = 0.442, *p* = 0.000) is statistically significant. The second direct positive hypothesis support (β = 0.348, *p* = 0.000) to students’ engagement is likewise substantial. The third direct hypothesis linking students’ engagement to CL (β = 0.089, *p*-value = 0.143) is not significant but significant at 0.1. Another relationship proposed is the moderating influence of group size on the link between peer support and student engagement. According to the results, a positive and statistically significant interaction between group size and peer support impacts students’ engagement (β = 0.209; *p* = 0.000). Table 9 represents the specific indirect impact. The association between CL and students’ engagement is mediated by peer support (β = 0.154; *p* = 0.000). The coefficient’s determinant represents the quality of model fit. The percentage represents the variance in the dependent variables caused by the independent factors. *R*^2^ helps to explain the model’s variance. *R*^2^, which describes the regressive nature of the proposed correlations, is a statistical method. *R*^2^ = 0.195, indicating that CL has a 19.5% variance in peer support. Student engagement’s correlation value (*R*^2^) is 0.332, indicating a 33.2% variance. Chin (2010) performed research with a modest *R*^2^ value. The predictive accuracy criterion, called *Q*^2^, indicates the model’s predictive significance. Geisser (2017) describes it as Stone-Geisser’s *Q*^2^. Specifically, *Q*^2^ must be greater than zero. A number below zero indicates that the dependent variables are insufficient to account for the variance. Peer support and student engagement were measured in the current research at *Q*^2^ values of 0.126 and 0.229. In this study, *Q*^2^ surpasses zero. Predictions that describe the model are thus relevant.

**TABLE 8:** Direct structural paths.

	Coefficient	Standard deviation	*T* values	*p-*Values
Collaborative learning peer support	0.442	0.051	8.592	0.000
Collaborative learning students’ engagement	0.113	0.062	1.805	0.071
Group size students’ engagement	0.220	0.042	5.259	0.000
Peer support students’ engagement	0.348	0.042	8.298	0.000
Group size × peer support students’ engagement	0.209	0.040	5.192	0.000

**TABLE 9:** Specific indirect effect.

	Coefficient	Standard deviation	*T*-value	*p*-Value
Collaborative learning peer support students engagement	0.154	0.025	6.059	0.000

## 5 Discussion and conclusion

The study’s results provide important insights into the intricate links between CL, peer support, group size, and student engagement (Kebede et al., 2024). The study found that CL strongly impacted peer support, with a coefficient of 0.442 (*p* < 0.001). This shows that engaging students in collaborative activities promotes a strong feeling of mutual support among peers, consistent with previous research (Patrick et al., 2007) stressing the social advantages of collaborative educational approaches. However, the direct impact of CL on student engagement was not statistically significant, with a coefficient of 0.113 (*p* = 0.071), showing that CL alone may not be enough to increase engagement without other supporting mechanisms (Herrmann, 2013). A significant correlation of 0.348 (*p* < 0.001) suggests that peer support is key to increasing student engagement. This research emphasizes the critical role of helpful peer relationships in keeping students motivated and engaged in learning activities (Urdan and Schoenfelder, 2006). The research found a significant indirect path (coefficient = 0.154, *p* < 0.001), indicating that peer support acts as a mediator between CL and student engagement. This demonstrates that the positive influence of CL on engagement is mostly manifested via the supportive social environment it fosters among peers (Chen et al., 2018). The study revealed a substantial moderating influence of group size on the connection between peer support and student engagement, with a coefficient of 0.209 (*p* < 0.001). The positive interaction effect shows that the impact of peer support on engagement grows with group size, perhaps owing to the higher variety of contacts and resource-sharing possibilities in bigger groups (Qi et al., 2023; Tang and Hew, 2022). However, this impact most likely depends on efficient group communication and coordination. Theoretically, these results improve our knowledge of CL techniques by focusing on the social aspects that influence engagement. In practice, they recommend that instructors aggressively promote peer support in collaborative contexts and carefully limit group size to maximize engagement results (Van Ryzin and Roseth, 2018). While the research offers insight into crucial processes, the fact that CL had no significant direct influence on engagement suggests that other mediating or contextual variables, such as task difficulty or intrinsic motivation, should be investigated further. Future research might also benefit from longitudinal designs to investigate how these associations grow over time and if their effects persist across learning environments.

### 5.1 Implications

These results have implications for better teaching, learning, and peer support. Students may improve and grow their English skills beyond intermediate if instructors develop and execute high-quality CL activities, push students to value the assignments and provide strong peer support. Furthermore, the findings indicated that peer support and group size were critical in increasing students’ engagement in the English curriculum via CL activities. This study’s findings have important implications for theory and practice regarding English programs in Chinese public colleges. The established connection between CL and increased peer support indicates that integrating structured group activities into the curriculum can create an environment that promotes both academic and emotional support among students. Peer interactions are essential in language learning, significantly enhancing communication skills and overall engagement. The mediating role of peer support clarifies how CL affects student engagement. This insight emphasizes the importance of understanding CL as a multifaceted social process that promotes both cognitive and emotional growth. Future research should investigate the relationship between group dynamics and learning outcomes, incorporating additional variables such as cultural influences and task complexity.

Educators can implement various strategies to effectively apply these findings to enhance teaching practices. Teachers should create collaborative tasks that explicitly encourage peer interaction and support, such as implementing role assignments or rotating leadership within small groups. Data indicate the moderating influence of group size; therefore, educators should consider utilizing smaller groups to improve interpersonal communication, while also acknowledging the advantages of larger groups for tasks that necessitate diverse inputs. Furthermore, training sessions or workshops focused on effective communication and conflict resolution can enhance students’ ability to optimize CL experiences. The incorporation of regular peer feedback sessions can enhance the support system within the classroom. These sessions must be organized to deliver constructive feedback and foster self-reflection, thus facilitating both academic and personal development. In conclusion, based on practical teaching experiences, institutions ought to establish environments that incentivize collaborative success and acknowledge the contributions of students who enhance group learning dynamics. Initiatives may encompass peer mentoring programs and collaborative project showcases that emphasize successful group interactions.

This study highlights the importance of CL in developing strong peer support networks, which significantly improve student engagement. Implementing these strategies enables educators to develop dynamic and supportive learning environments that promote academic success and enhance essential social and communicative skills.

### 5.2 Limitations and future directions

While this research sheds light on the links between CL, peer support, group size, and student engagement, numerous limitations require consideration. First, although the research looks at the moderating influence of group size, it does not take into consideration other potentially relevant group features like group makeup, cohesion, or diversity, which may affect peer interactions and learning outcomes. Future study should look at these factors to acquire a better understanding of group dynamics in CL settings. Furthermore, although the assessment instruments employed in this research were modified from previously validated measures, they may not completely reflect the complex character of notions such as peer support and CL in the context of English programs at Chinese public universities. Furthermore, although the sample size was enough for SEM analysis, it came from a rather homogenous educational setting, which may restrict the results’ applicability to other programs or institutions.

Future studies might address these limitations by refining the study methodology, including additional group characteristics, and expanding the inquiry to various educational contexts to improve the results’ robustness and application. Regarding the study’s shortcomings, we accept that the limited number of participants limits the results’ generalizability. The research was conducted in Chinese colleges; however, we utilized a bootstrapping procedure with 5,000 resamples to ensure absolute universality. We utilized solely second-year college students. This restricts the generalizability of the conclusions outside this group, as shown in China and elsewhere (education board). This allows future studies to explore a wider sample of programs, higher education institutions, and undergraduate levels. We used several scales, answer formats, and three waves of data collection with a 3-week time lag to decrease typical technique bias. However, we propose utilizing stronger measures, such as a longitudinal approach, for future investigations. Although this research was done in China, the topics highlighted apply to other nations.

## Data Availability

The original contributions presented in this study are included in this article/supplementary material, further inquiries can be directed to the corresponding author.
